# Improving Ex Vivo Nasal Mucosa Experimental Design for Drug Permeability Assessments: Correcting Mucosal Thickness Interference and Reevaluating Fluorescein Sodium as an Integrity Marker for Chemically Induced Mucosal Injury

**DOI:** 10.3390/ph18060889

**Published:** 2025-06-13

**Authors:** Shengnan Zhao, Jieyu Zuo, Marlon C. Mallillin, Ruikun Tang, Michael R. Doschak, Neal M. Davies, Raimar Löbenberg

**Affiliations:** 1 Faculty of Pharmacy and Pharmaceutical Sciences, University of Alberta, Edmonton, AB T6G 2E1, Canada; shengna2@ualberta.ca (S.Z.); zjieyu@ualberta.ca (J.Z.); mallilli@ualberta.ca (M.C.M.III); ruikun3@ualberta.ca (R.T.); mdoschak@ualberta.ca (M.R.D.); 2 China Z. Pharmaceutical Productivity Centre, Beijing 101111, China; 3 Department of Pharmacy, Faculty of Pharmacy, University of Santo Tomas, Manila 1015, Philippines

**Keywords:** nasal mucosa, thickness correction, nasal permeability, Franz cell, ex vivo permeation study, fluorescein sodium, simulation modeling

## Abstract

**Objectives**: Ex vivo nasal mucosa models provide physiologically relevant platforms for evaluating nasal drug permeability; however, their application is often limited by high experimental variability and the absence of standardized methodologies. This study aimed to improve experimental design by addressing two major limitations: the confounding effects of mucosal thickness and the questionable reliability of fluorescein sodium (Flu-Na) as an integrity marker for chemically induced mucosal injury. **Methods**: Permeability experiments were conducted using porcine nasal tissues mounted in Franz diffusion cells, with melatonin and Flu-Na as model compounds. Tissues of varying thickness were collected from both intra- and inter-individual sources, and a numerical simulation-based method was employed to normalize apparent permeability coefficients (Papp) to a standardized mucosal thickness of 0.80 mm. The effects of thickness normalization and chemically induced damage were systematically evaluated. **Results**: Thickness normalization substantially reduced variability in melatonin Papp, particularly within same-animal comparisons, thereby improving statistical power and data reliability. In contrast, Flu-Na exhibited inconsistent correlations across different pigs and failed to reflect the expected increase in permeability following isopropyl alcohol (IPA)-induced epithelial damage. These results suggest that the relationship between epithelial injury and paracellular transport may be non-linear and not universally applicable under ex vivo conditions, limiting the suitability of Flu-Na as a standalone marker of mucosal integrity. **Conclusions**: The findings highlight the importance of integrating mucosal thickness correction into standardized experimental protocols and call for a critical reassessment of Flu-Na in nasal drug delivery research.

## 1. Introduction

The permeability of candidate compounds is a critical determinant of drug development, influencing both druggability and bioavailability. In the 1990s, approximately 30–40% of investigational new drugs failed during preclinical and clinical stages due to inadequate biopharmaceutical properties [1,2]. Advances in in vitro permeability screening and formulation strategies subsequently reduced this failure rate to 10–15% by the early 2000s [1,2]. Therefore, early and accurate permeability assessment of new chemical entities is essential for optimizing lead candidates, improving success rates, and facilitating clinical translation.

Nasal drug delivery has gained increasing attention as a non-invasive route that bypasses first-pass metabolism and enables rapid systemic and central nervous system absorption [3]. However, the unique physiological characteristics of the nasal cavity pose significant challenges to drug absorption. The nasal mucosal surface area (150–200 cm^2^) [4,5] is much smaller than that of the gastrointestinal tract (~32 m^2^) [6] or even the stomach (~530 cm^2^) [7], inherently limiting the available absorption interface. In addition, the nasal cavity contains only ~15 mL of mucus [8], restricting the dissolution capacity, particularly for poorly soluble compounds. The mucociliary clearance system further complicates absorption by continuously transporting the mucus layer toward the nasopharynx, where it is eventually swallowed into the gastrointestinal tract [9]. In healthy individuals, complete renewal of the mucus carpet typically occurs every 20–30 min [10], substantially reducing drug residence time and narrowing the window for effective nasal absorption.

Given these challenges, assessing drug permeability under physiologically relevant nasal conditions is essential for evaluating the feasibility of nasal drug delivery [11,12]. Traditionally, permeability assessment has relied on in vitro models such as the parallel artificial membrane permeability assay (PAMPA) and various cell-based systems.

The PAMPA is a high-throughput screening tool initially developed to evaluate the passive diffusion permeability of oral drugs [13]. With continued advancements, modified versions of PAMPA have been adapted for various biological barriers, including nasal permeability (Nasal-PAMPA [14]), the blood–brain barrier (BBB-PAMPA [15]), skin (Skin-PAMPA [16]), and the cornea (Corneal-PAMPA [17]). The nasal-PAMPA model, which incorporates 0.5% (*w*/*v*) mucin in the donor compartment and 2% (*w*/*v*) phosphatidylcholine in the lipid membrane, has been used to differentiate between highly and poorly permeable compounds under simulated nasal conditions [14]. However, a major limitation of PAMPA is that it assesses only passive diffusion and does not account for active transport processes or enzymatic metabolism, both of which are critical for understanding nasal drug absorption.

Cell-based models offer an alternative approach to permeability assessment. While the Caco-2 cell line is widely used to predict oral drug absorption [18], nasal epithelial cell lines such as RPMI 2650 and Calu-3 are more relevant for nasal drug delivery due to their closer physiological resemblance to the nasal and respiratory epithelium [19]. RPMI 2650, derived from squamous cell carcinoma, has been recognized as a predictive in vitro model for nasal permeability studies [20]. However, a single immortalized nasal epithelial cell line cannot fully replicate the diversity of cell types present in the pseudostratified ciliated columnar epithelium found in nasal mucosa [20], such as goblet cells and basal cells, which play crucial roles in mucus production and barrier function. These limitations restrict the ability of current cell culture models to comprehensively predict nasal drug permeability.

The ex vivo nasal mucosa model retains the complex cellular composition of the native nasal barrier, thereby better preserving its physiological functions. As a result, it provides a more physiologically relevant alternative to immortalized cell lines for studying nasal drug permeability and absorption mechanisms. This model enables direct assessment of drug permeability using excised nasal tissues, offering higher predictive accuracy for human absorption. In addition, utilizing tissues from animals approved for human consumption minimizes unnecessary animal euthanization, aligning with ethical considerations in drug development. However, species differences in nasal anatomy and epithelial composition can significantly affect drug absorption predictions, making the choice of an appropriate animal model critical. Large animals such as pigs and sheep possess nasal mucosal structures closely resembling those of humans, rendering them more suitable for ex vivo permeability studies and enhancing translational relevance [21,22,23,24].

Despite these advantages, ex vivo tissue models are subject to considerable variability due to differences in tissue thickness and metabolic activity, and standardized protocols for ex vivo nasal permeability studies remain underdeveloped. Establishing standardized experimental protocols is crucial to ensure reliable and reproducible permeability assessments.

To improve the reliability and standardization of ex vivo nasal mucosa permeability studies, this study evaluates the effectiveness of a simulation-based approach for reducing variability in apparent permeability coefficient (Papp) measurements. In addition, fluorescein sodium (Flu-Na) is reassessed as an integrity marker for chemically induced mucosal injury. Together, these evaluations aim to enhance the reliability and interpretability of ex vivo permeability data and to support the development of standardized, mechanism-informed protocols for intranasal drug delivery research.

## 2. Results and Discussion

### 2.1. The Influence of Mucosal Thickness on Intra- and Inter-Individual Variability in Nasal Permeability

Ensuring data reproducibility and cross-sample comparability remains a persistent challenge in ex vivo nasal permeability studies, primarily due to the inherent variability of biological tissues [25]. Among the various sources of experimental variation, mucosal thickness has emerged as a particularly influential confounding factor, capable of masking true permeability differences between tested compounds [26]. Since ex vivo drug permeation is predominantly governed by passive diffusion, this relationship is well described by Fick’s law, which states that diffusion flux is inversely proportional to barrier thickness [27]. Thus, mucosal thickness represents a critical determinant of drug permeation. When tissue thickness varies between samples, especially without appropriate correction, permeability comparisons may become misleading or difficult to interpret.

**Figure 1:**
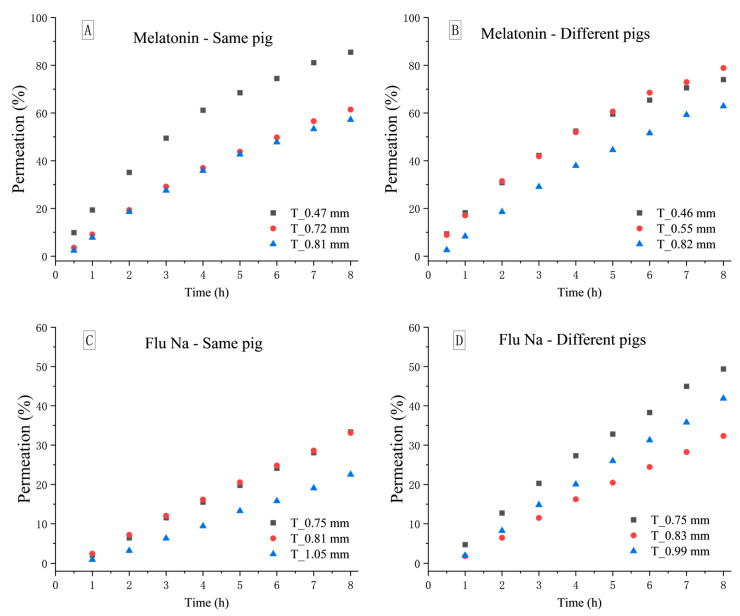
Intra- and inter-individual variability in melatonin and Flu-Na permeability across porcine nasal mucosa. Permeation profiles of melatonin (**A**,**B**) and Flu-Na (**C**,**D**) over an 8 h ex vivo diffusion study using porcine nasal mucosa. Left panels represent intra-individual comparisons (same pig, n = 3), while right panels represent inter-individual comparisons (different pigs, n = 3). Each data set includes mucosal tissues of three different thicknesses: black squares (thinnest tissue), red circles (intermediate thickness), and blue triangles (thickest tissue). T values indicate the measured mucosal thickness for each sample. Permeation (%) was calculated as the cumulative percentage of the initial donor concentration over time.

As shown in Figure 1, distinct differences in permeation profiles were observed for both melatonin and Flu-Na across nasal tissues of varying thickness. For melatonin, a clear inverse relationship between tissue thickness and permeability was evident in intra-individual comparisons (Figure 1A), where the thinnest mucosa consistently exhibited the highest permeation. A similar trend was observed across different pigs (Figure 1B); however, when thickness differences were minimal, the expected correlation was occasionally masked or even reversed due to inter-individual biological variability. Nevertheless, when thickness differences were sufficiently pronounced, melatonin permeability reliably declined with increasing tissue thickness.

In contrast, the relationship between tissue thickness and permeability was less consistent for Flu-Na. In intra-individual comparisons (Figure 1C), only a weak inverse correlation was observed, while in inter-individual comparisons (Figure 1D), the association between thickness and Papp was almost entirely obscured. These findings suggest that for highly hydrophilic compounds relying predominantly on paracellular transport, biological differences in mucosal microstructure—such as epithelial composition, tight junction expression, or vascular density—may outweigh the impact of barrier thickness itself.

These findings highlight that although mucosal thickness is a key determinant of drug permeability, its influence may be attenuated or masked when thickness differences are minimal, particularly in the presence of substantial intra- or inter-individual biological variability. Notably, the variability observed for Flu-Na was greater than that for melatonin, suggesting that hydrophilic compounds relying predominantly on paracellular transport may be more sensitive to subtle tissue-level heterogeneity. This heightened susceptibility raises concerns regarding the reliability of Flu-Na as a marker of epithelial or paracellular integrity. Furthermore, the lack of a consistent correlation between mucosal thickness and permeability in the inter-individual Flu-Na data underscores the complexity of interpreting permeability outcomes under conventional ex vivo conditions.

In our previous work, we developed a numerical simulation-based method to normalize permeability data to a standardized mucosal thickness [26], which effectively reduced variability among samples collected from the same animal. In the present study, this approach was extended to evaluate whether it could also reduce variability associated with mucosal thickness differences across tissues obtained from different animals. Two model compounds—melatonin and Flu-Na—were selected to represent distinct physicochemical properties, and mucosal tissues were collected for both intra- and inter-individual comparisons.

**Figure 2:**
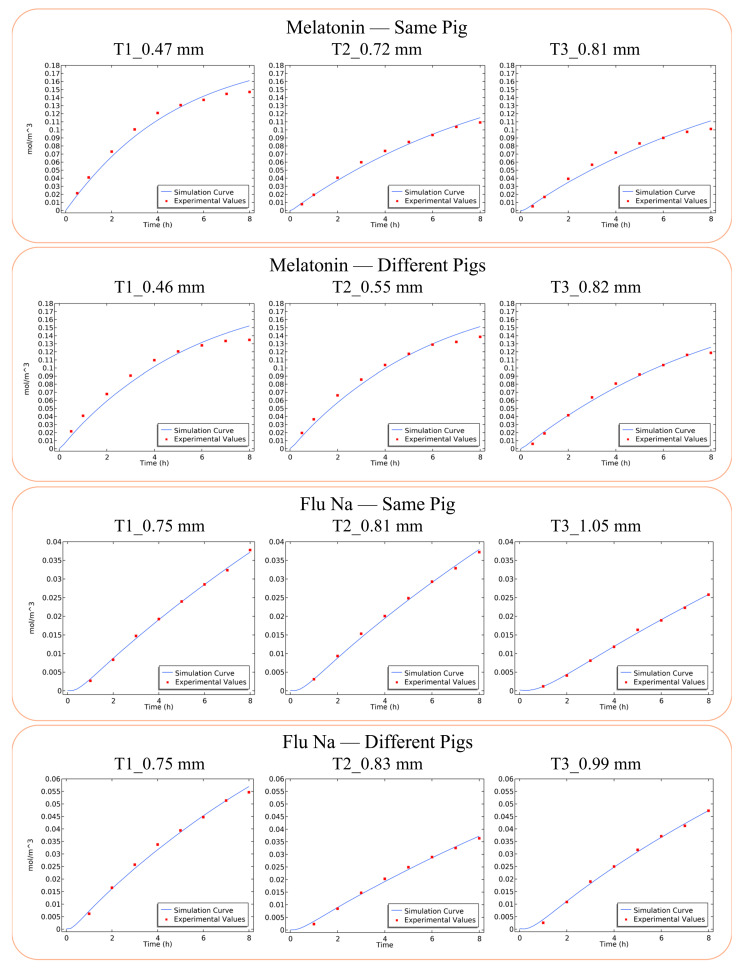
Comparison of simulated and experimental permeation profiles of melatonin and Flu-Na across porcine nasal mucosa. Simulated (blue lines) and experimental (red dots) permeation profiles of melatonin and Flu-Na are presented over an 8 h ex vivo Franz diffusion study.

**Figure 3:**
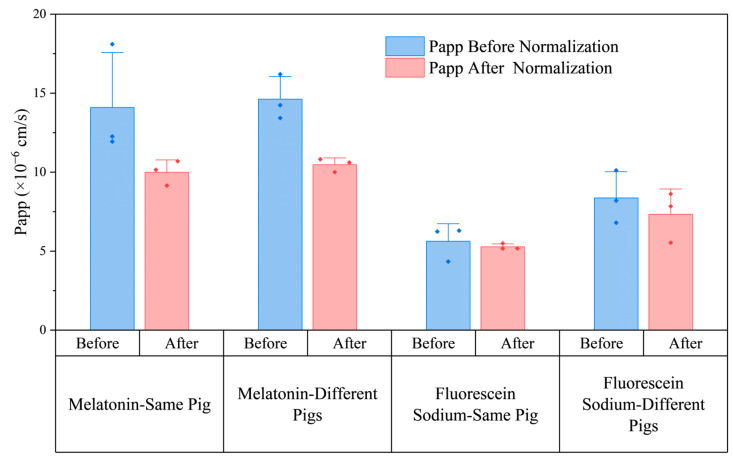
Effect of thickness normalization on apparent permeability (Papp) across porcine nasal mucosa. Mean Papp values before and after thickness normalization for melatonin and Flu-Na in intra- and inter-individual porcine nasal tissues. Normalization was performed using a simulation model with a reference mucosal thickness of 0.80 mm. Error bars represent standard deviations (n = 3), individual data points are shown as dots.

The established simulation model was adapted to the current experimental settings by incorporating actual experimental parameters, including measured mucosal thickness, donor and receptor chamber volumes, and the initial loading concentration. Permeation profiles obtained from the Franz diffusion studies were fitted using the numerical model to generate permeability parameters characterizing drug transport behavior under a standardized thickness. The consistency between experimental and simulated curves is shown in Figure 2. The estimated parameter values derived from the simulation are provided in Supplementary Data Table S1, and detailed theoretical definitions and mathematical assumptions are available in our previous work [26]. Using this simulation approach, the Papp of each sample was normalized to a reference mucosal thickness of 0.80 mm. Papp values before and after thickness normalization are compared in Figure 3 to illustrate the effectiveness of the model in reducing intra- and inter-sample variability.

The agreement between experimental and simulated permeation profiles (Figure 2) was generally better for thicker mucosal tissues, whereas greater deviations were observed in samples with thinner membranes. This discrepancy may be attributed to the greater fragility of thinner mucosa, making it more susceptible to structural or functional degradation during the diffusion study, which could alter permeability characteristics over time.

As shown in Figure 3, the mean Papp values of melatonin and Flu-Na before and after mucosal thickness normalization are presented for both intra- and inter-individual porcine nasal tissues. After normalization to a standardized thickness of 0.80 mm, the mean Papp values for melatonin were highly consistent between the same-pig and different-pig groups, both before (14.10 ± 3.47 × 10^−6^ cm/s vs. 14.62 ± 1.42 × 10^−6^ cm/s) and after normalization (10.00 ± 0.78 × 10^−6^ cm/s vs. 10.48 ± 0.42 × 10^−6^ cm/s), suggesting that inter-individual variability was minimal once thickness was accounted for. For Flu-Na, the inter-individual group exhibited a higher mean Papp than the intra-individual group after normalization (7.33 ± 1.60 × 10^−6^ cm/s vs. 5.28 ± 0.18 × 10^−6^ cm/s), although this difference did not reach statistical significance.

**Figure 4:**
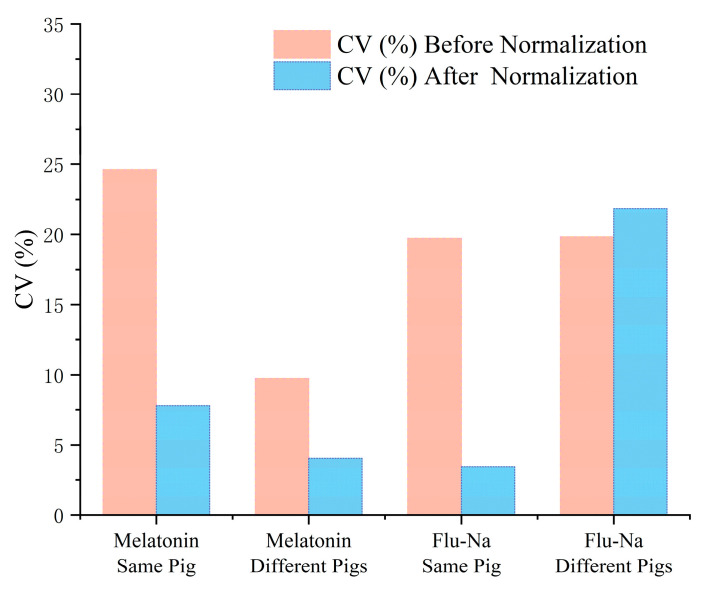
Effect of thickness normalization on the coefficient of variation (%) of apparent permeability (Papp) measurements.

Figure 4 further illustrates the effect of thickness normalization on the variability of Papp estimates, as measured by the coefficient of variation (%). For melatonin, normalization substantially reduced variability in both intra- and inter-individual groups, with CV decreasing by 16.8% in the same-pig group and by 5.7% in the different-pig group. These results confirm that mucosal thickness was a primary contributor to experimental variability for melatonin and that simulation-based normalization effectively enhanced data consistency. In contrast, the response to normalization was compound dependent. For Flu-Na, normalization successfully reduced intra-individual variability (CV 19.72% → 3.43%) but failed to reduce variability in the inter-individual group, where CV paradoxically increased (19.83% → 21.85%).

Although simulation-based normalization effectively reduced variability for melatonin and partially for Flu-Na, mucosal thickness alone does not fully account for the experimental variation observed. Several additional biological and procedural factors may influence ex vivo permeability outcomes. The anatomical region plays a critical role, as differences in epithelial architecture, vascularization, glandular activity, and metabolic function may differentially affect the transport of hydrophilic and lipophilic compounds [25,28,29]. Tissue freshness is another key factor, as physiological activity such as ciliary motion will cease post mortem after excision, potentially impairing barrier function during extended experimental durations [30]. Storage conditions prior to testing also impact tissue integrity; for instance, mild epithelial disruption may occur after short-term refrigeration (24–48 h), while prolonged freezing (e.g., −20 °C for several weeks) often leads to significant structural damage and exaggerated permeability values [31]. These biological and procedural factors may help explain why Flu-Na, a highly hydrophilic compound dependent on paracellular pathways, exhibited substantial inter-individual variability even after normalization, suggesting heightened sensitivity to subtle microstructural heterogeneity.

### 2.2. Statistical Efficiency After Mucosal Thickness Normalization

In permeability studies, statistical efficiency—defined as the ability to detect meaningful differences with a minimal number of replicates—is critically dependent on inter-sample variability. High variability not only obscures true permeability differences but also increases the number of samples required to achieve statistical significance. This issue is particularly pronounced in ex vivo studies, where tissue availability is inherently limited. To minimize variability, researchers often rely on mucosal samples harvested from the same animal to reduce biological noise and improve comparability. However, the quantity of tissue obtainable from a single individual is typically insufficient to support multi-compound screening or replicate testing. Increasing the number of donor animals to obtain more tissue introduces additional inter-individual variability, which can confound interpretation and diminish statistical power.

Therefore, implementing a strategy to reduce experimental variability—such as mucosal thickness normalization—is essential not only for improving mechanistic clarity but also for enhancing the statistical robustness of experimental outcomes, particularly in studies constrained by limited tissue availability.

From a statistical design perspective, the minimum detectable relative difference between two groups in a two-tailed independent *t*-test can be estimated as described in Section 3.5 (Equation (1)). Table 1 illustrates the practical implications of this relationship, presenting the minimum relative differences required to achieve statistical significance under various combinations of CV and sample size. Notably, reducing variability yields a more substantial improvement in detection sensitivity than merely increasing sample size. For example, at a CV of 30%, increasing the number of replicates from 3 to 5 lowers the detectable difference from 68.0% to 52.67%. However, maintaining n = 3 and reducing CV from 30% to 20% results in a more pronounced improvement, with the threshold dropping to 45.33%. Further reducing CV to 10% enables detection of differences as small as 22.67% with the same sample size. These results demonstrate that reducing variability—such as through mucosal thickness normalization—is more effective than simply increasing sample size when aiming to enhance detection sensitivity in ex vivo permeability studies.

This effect was particularly evident in the melatonin datasets of the present study, where mucosal thickness normalization reduced the CV from 24.61% to 7.79% in intra-individual comparisons and from 9.74% to 4.04% in inter-individual groups. These reductions translated into substantial improvements in statistical efficiency, enabling the same level of power to be achieved with fewer replicates or allowing the detection of smaller intergroup differences at a fixed sample size. In contrast, previous ex vivo studies that did not account for mucosal thickness frequently reported high inter-sample variability. For example, in porcine respiratory mucosa, mannitol exhibited CVs ranging from 59.3% to 62.6%, while testosterone showed even greater variability, with CVs between 93.7% and 106.9% [32]. In another study using bovine nasal tissues, dimenhydrinate formulations delivered via self-emulsifying drug delivery systems resulted in CVs ranging from 8.4% to 24.6% [33]. Similarly, permeability of the beta-blocker drug atenolol across sheep nasal mucosa showed a CV of 16.42% [34]. Collectively, these data highlight the substantial variability that can arise in the absence of structural normalization, underscoring the need for thickness correction in comparative permeability studies.

These findings confirm that mucosal thickness normalization not only improves statistical robustness but also enhances the interpretability of nasal drug permeability data, particularly in ex vivo systems constrained by limited tissue availability. By systematically reducing biological variability, normalization enables more reliable compound comparisons and more efficient study designs, thereby supporting both mechanistic and translational research objectives.

### 2.3. Reevaluating Flu-Na as an Integrity Marker for Chemically Induced Mucosal Injury

**Figure 5:**
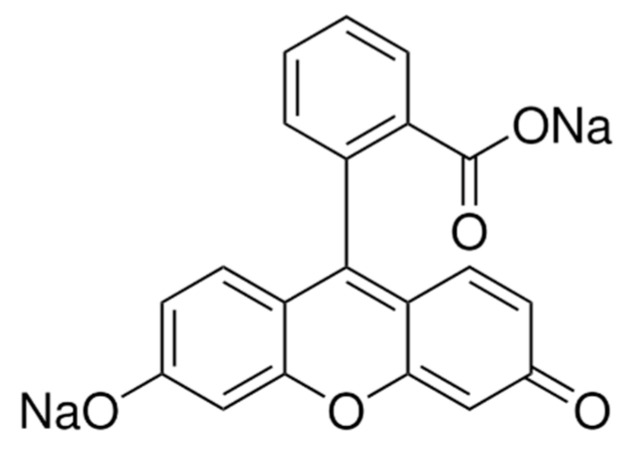
Chemical structure of Flu-Na.

Flu-Na is a small organic dye featuring a xanthene ring core with carboxyl and hydroxyl substituents, which confer strong hydrophilicity. Its chemical structure is shown in Figure 5. Despite its known hydrophilicity, reported log P values for Flu-Na vary considerably in the literature, ranging from −0.67 to −1.52 [35,36]. Computational predictions using ADMET Predictor version 11.0 (Simulations Plus, Inc., Lancaster, CA, USA; https://www.simulations-plus.com/software/admetpredictor/, accessed on 26 March 2025) yielded positive log D values of 4.17 at pH 6.0 and 2.00 at pH 10.0, both inconsistent with the expected hydrophilic properties of Flu-Na. Such discrepancies raise concerns regarding the reliability of existing data and whether they accurately reflect the physicochemical characteristics of Flu-Na.

**Figure 6:**
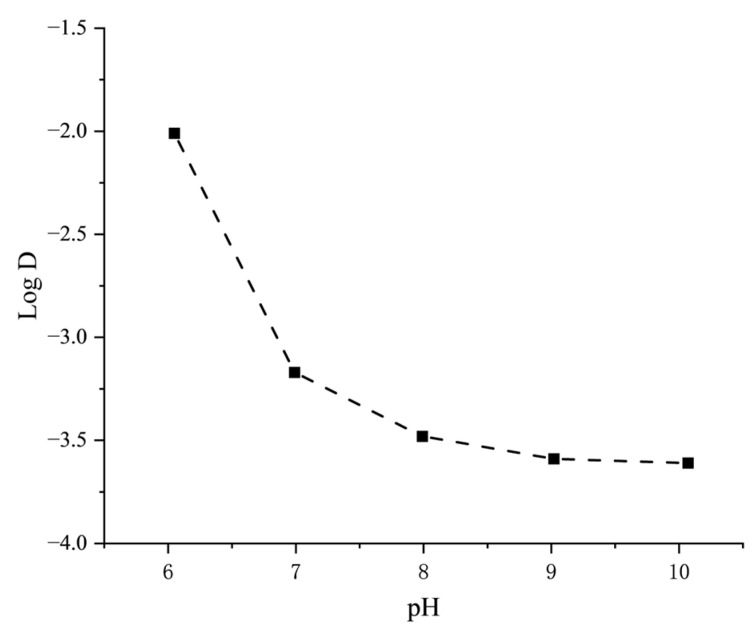
Experimentally determined log D values of Flu-Na at different pH conditions using the shake-flask method.

To address this uncertainty, the log D values of Flu-Na were experimentally determined using a shake-flask method following standard partitioning procedures (Figure 6). The measured log D values exhibited a clear pH dependency: as the pH increased from 6.0 to 10.0, the log D values became progressively more negative, confirming the highly hydrophilic nature of Flu-Na under physiological and slightly alkaline conditions.

**Figure 7:**
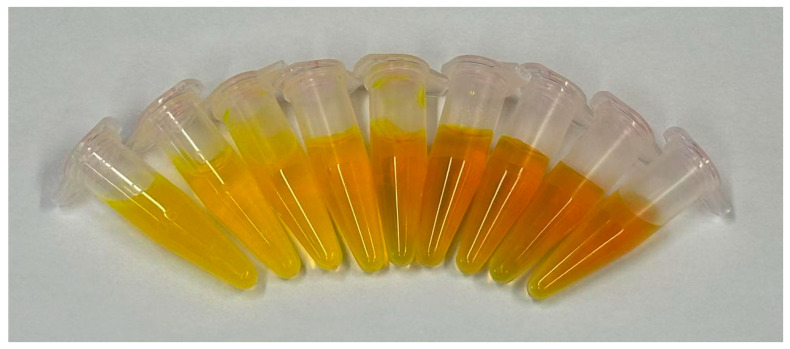
Visual appearance of Flu-Na solutions (1 mg/mL) at different pH values. Solutions were prepared at a concentration of 1 mg/mL, and visual appearance was recorded after pH adjustment. From left to right, the pH values are 4.85 (precipitation observed), 5.6, 6.0, 7.0, 8.0, 9.0, 10.0, 11.0, and 12.0.

**Figure 8:**
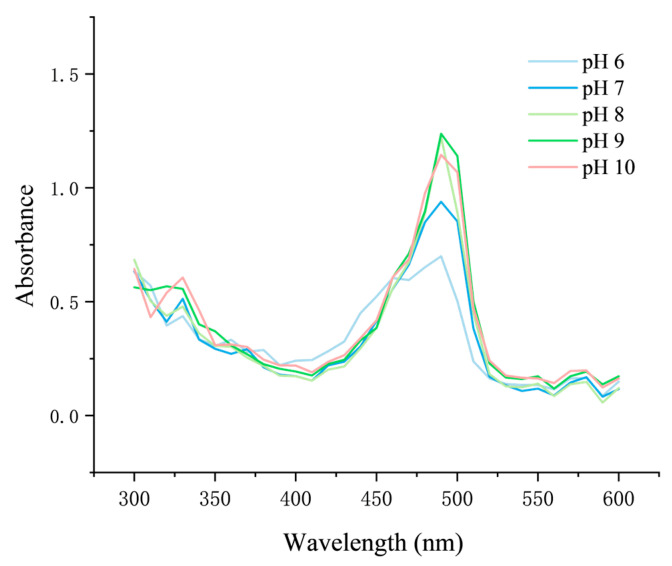
UV-visible absorption spectra of Flu-Na solutions at different pH values (300–600 nm). UV-visible spectra of Flu-Na solutions (20 µg/mL) were recorded across the 300–600 nm range.

Further characterization revealed that Flu-Na exists predominantly in an ionized form under physiological conditions, with its degree of protonation varying with pH and significantly affecting its log D values. As the pH increased from 6.0 to 10.0, deprotonation enhanced hydrophilicity, resulting in progressively more negative log D values. These protonation-dependent structural changes also influenced the solution color (Figure 7) and the UV-visible absorption spectra (Figure 8). Spectral scans across the 300–600 nm range revealed pronounced absorbance differences at the peak wavelength. Therefore, for accurate quantification, all measurements were conducted at the same pH to ensure consistency. Previous studies similarly reported that the log P of fluorescein decreased from approximately 2.78 at pH 4.29 to −0.08 at pH 7.5 [37], supporting the pH-dependent shift in partitioning behavior observed for Flu-Na.

Given the confirmed hydrophilicity and pH-dependent behavior of Flu-Na, its utility as a paracellular permeability marker was further evaluated. Flu-Na is widely used in cell culture models to assess tight junction integrity. In Caco-2 monolayers, confocal microscopy has revealed its predominant localization within the intercellular space, establishing its role in evaluating paracellular transport [36,38]. Increased Flu-Na permeability reflects paracellular leakage and barrier disruption and is often paired with transepithelial electrical resistance measurements to monitor cellular barrier development. This well-established correlation supports its broad application as a surrogate marker of epithelial integrity in studies of drug absorption, permeability testing, and toxicology screening.

In addition to cell culture models, Flu-Na has also been applied in various in vivo systems to assess epithelial and mucosal barrier integrity. In animal models, Flu-Na is regarded as one of the most sensitive and early indicators of blood–brain barrier disruption, owing to its small molecular size and paracellular transport characteristics [39]. Similarly, in a mouse intestinal ischemia–reperfusion model, Flu-Na has been validated as a dynamic tracer of mucosal permeability, with increased tissue accumulation correlating with epithelial injury [40]. In ophthalmic research, in vivo fluorometric assessment of corneal permeability further supports the use of Flu-Na as a quantitative marker of epithelial barrier function [41].

Furthermore, Flu-Na has been widely adopted in ex vivo permeability studies using various animal-derived mucosal tissues to evaluate the barrier-modulating effects of different compounds and excipients, including guinea pig intestinal mucosa [42,43], rabbit cornea [44], and porcine buccal mucosa [45].

However, despite its widespread applications, the reliability of Flu-Na under chemically induced epithelial injury conditions remains underexplored. In a preliminary study using 10% isopropyl alcohol (IPA) as a positive control [46] to induce epithelial damage, a paradoxical decrease in Flu-Na permeability (Papp) was observed compared to tissues treated with DPBS as a negative control (unpublished data). Since the two groups were derived from different donor pigs, and considering the substantial inter-individual variability previously observed in Flu-Na permeability, no definitive conclusion could be drawn.

**Figure 9:**
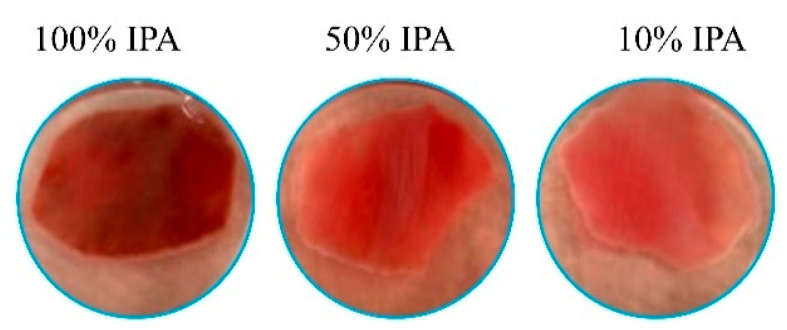
Representative images of porcine nasal mucosa following exposure to graded concentrations of isopropyl alcohol (IPA). Visual differences in tissue appearance after 30 min immersion in increasing concentrations of IPA, indicating varying levels of epithelial disruption.

**Figure 10:**
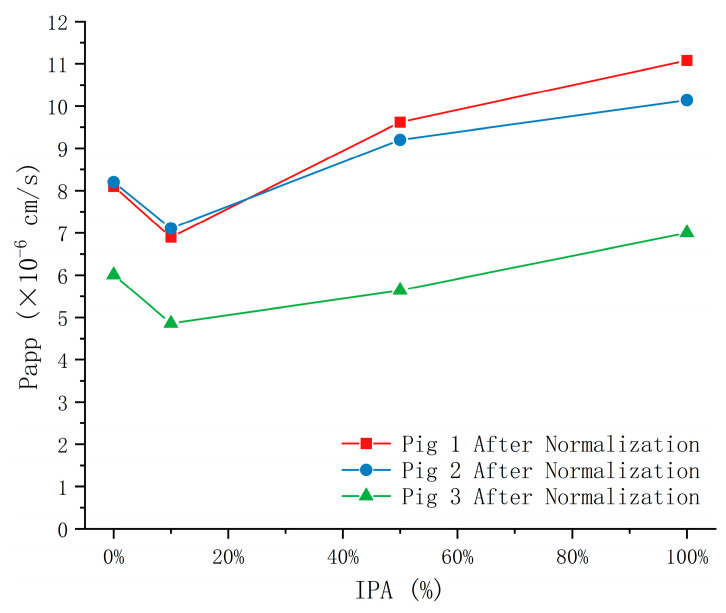
Effect of isopropyl alcohol (IPA) on Flu-Na permeability across porcine nasal mucosa after thickness normalization.

To systematically investigate this issue, nasal mucosa tissues harvested from the same pig were immersed in 10%, 50%, or 100% IPA for 30 min to induce graded levels of chemical damage. A parallel control sample was incubated in DPBS under identical conditions. Flu-Na permeability was then measured as an indicator of mucosal integrity. This experiment was independently repeated in tissues from three different pigs to ensure reproducibility. Representative images of nasal mucosa following IPA-induced damage are shown in Figure 9, and the corresponding Papp values, normalized to a standardized mucosal thickness of 0.80 mm, are presented in Figure 10.

Unexpectedly, the permeability results diverged from initial expectations: nasal mucosa treated with 10% IPA exhibited lower Flu-Na permeability than tissues incubated in DPBS as a negative control. Although a general trend of increasing Papp values was observed with escalating IPA concentrations (10%, 50%, and 100%), the initial reduction at low concentration challenges the conventional assumption that epithelial damage universally enhances paracellular permeability. These findings suggest that the relationship between epithelial injury and paracellular transport is likely non-linear and may not apply uniformly under ex vivo conditions. These results echo prior observations in studies using different permeation enhancers across ex vivo mucosal models, where compound-specific and barrier-specific interactions could lead to counterintuitive permeability outcomes [12].

One plausible explanation for this paradox lies in the nature of IPA-induced tissue damage. At lower concentrations, IPA may cause epithelial dehydration and induce conformational or functional alterations in membrane-associated proteins, leading to functional tightening rather than structural disruption of the barrier. Tight junctions, composed of transmembrane proteins such as occludin, claudins, and tricellulin, along with scaffold proteins like ZO-1, regulate paracellular transport by forming a dynamic barrier that transitions between open and closed states in response to physiological or chemical stimuli [47]. Disruption or modification of these regulatory proteins may shift the junctional complex toward a more restrictive configuration, thereby reducing the permeability of small hydrophilic molecules such as Flu-Na.

In contrast, higher concentrations of IPA are more likely to disrupt membrane lipids and cytoskeletal integrity, leading to epithelial exfoliation, tight junction protein delocalization, and increased paracellular leakage across the barrier. Histological studies support this dose-dependent disruption. For example, exposure of goat nasal mucosa to 100% IPA for one hour resulted in widespread epithelial loss and tissue injury [48], while similar damage was observed in sheep nasal tissue used in ciliary toxicity assays [49]. In guinea pigs, low-concentration IPA exposure induced reversible epithelial changes, whereas higher concentrations caused pronounced structural breakdown and functional impairment [50].

A comparable observation was reported in an ex vivo study using sheep nasal mucosa, where exposure to 5% *v*/*v* ethanol significantly reduced the permeability of Lucifer Yellow—a hydrophilic paracellular marker similar to Flu-Na. The authors concluded that the epithelial tissues were not damaged and that barrier permeability remained unchanged [51]. However, this interpretation may not be entirely accurate. It is possible that structural or biochemical alterations induced by ethanol exposure led to functional closure or occlusion of paracellular pathways, rather than preservation of a truly intact barrier.

In summary, these findings suggest that the structural consequences of chemical injury in ex vivo systems may not align with assumptions derived from classical in vitro models. The histological integrity of mucosal tissues plays a pivotal role in regulating paracellular flux, and epithelial injury does not invariably enhance the permeability of hydrophilic markers such as Flu-Na. Therefore, while Flu-Na remains a valuable tool for assessing epithelial integrity, caution should be exercised when interpreting its permeability changes under chemically induced injury conditions. A multiparametric approach—including histological evaluation, electrical resistance measurements, and the use of complementary permeability markers—is recommended for a more accurate assessment of mucosal barrier function.

### 2.4. Implications and Future Perspectives for Optimizing Ex Vivo Permeability Study Design

Variations in mucosal thickness have been clearly demonstrated to exert a profound impact on the calculated Papp, introducing substantial variability that can obscure true differences between compounds or treatment conditions [26]. To mitigate this confounding factor, thickness control must be considered a foundational aspect of ex vivo permeability study design. Two primary approaches can be employed: (1) selecting tissues with similar thicknesses at the experimental planning stage to minimize variability, or (2) applying post hoc mathematical normalization using simulation-based models to retrospectively correct for thickness-related deviations in Papp when uniform sampling is not feasible due to biological or logistical constraints.

In addition to correcting for mucosal thickness, our findings reveal that after normalization, inter-individual variability becomes markedly greater than intra-individual variability. This underscores the critical importance of performing parallel measurements using tissues from the same animal whenever possible to minimize biological variability and enhance data reproducibility. However, a practical limitation arises from the finite surface area available from a single mucosal specimen, which may not accommodate multiple replicates or simultaneous testing of several compounds.

To address this methodological challenge, we recommend prioritizing the use of tissues from the same individual for comparative studies. In parallel, we propose incorporating internal reference compounds with well-characterized permeability—preferably including both a high-permeability and a low-permeability probe—within each experimental set. By calculating the permeability of test compounds relative to these internal standards, a ratio-based normalization approach can be employed to mitigate system-level variability and enhance cross-sample comparability.

Furthermore, organic solvents with known mucosal toxicity should be avoided as formulation vehicles or media in permeability studies, as they can unpredictably alter mucosal permeability. Such changes are often non-linear and may contradict the common assumption that epithelial damage in ex vivo models consistently results in increased paracellular permeability.

Compared to conventional cell culture systems, the distinct behavior of Flu-Na in ex vivo mucosal tissues suggests that the mechanisms regulating paracellular transport in native mucosa may differ from assumptions based on monolayer models. Notably, our findings indicate that epithelial injury in biological tissues does not necessarily lead to increased permeability—it may also result in decreased permeability. This challenges the common practice of using elevated Flu-Na permeability as the sole indicator to rule out mucosal damage caused by solubilizers or excipients, as such assessments may be misleading. Interestingly, this property could also be harnessed to intentionally reduce mucosal permeability—for example, as a potential strategy to limit the entry of harmful or undesired substances across the epithelial barrier.

In the future, implementation of these strategies is expected to substantially improve the reproducibility of ex vivo permeability assays while maximizing tissue utilization and minimizing animal use. These methodological refinements also lay a technical foundation for the development of higher-throughput screening platforms to evaluate the mucosal permeability of drug candidates during early-stage formulation development.

## 3. Materials and Methods

### 3.1. Chemicals

Melatonin was purchased from Medisca Canada (Montreal, QC, Canada). Fluorescein sodium salt (CAS No. 518-47-8), Dulbecco’s phosphate-buffered saline (DPBS; containing MgCl_2_ and CaCl_2_), and HPLC-grade acetonitrile and water were obtained from Sigma-Aldrich (St. Louis, MO, USA). Isopropyl alcohol and *n*-octanol were acquired from Fisher Scientific (Nepean, ON, Canada). All other chemicals were of analytical grade.

### 3.2. Dissection and Thickness Measurement of Porcine Nasal Epithelial Tissues

Fresh disarticulated porcine heads (*Sus scrofa domesticus*) were obtained on the day of slaughter from a local meat supplier in Edmonton, Alberta. The pigs were raised for meat production, and the heads were transported to the laboratory immediately following humane slaughter. All tissues used in this study were derived from post-mortem specimens; no live animals were involved at any stage of the experimental process.

Nasal mucosa tissues were carefully dissected from the lower turbinates within the nasal cavity. Immediately after excision, the tissues were immersed in chilled DPBS to prevent desiccation and to preserve structural integrity. The samples were then cut into appropriate sizes, and mucosal thickness was measured using an electronic digital caliper (Fisher Scientific, Waltham, MA, USA) as described in previous work [26].

Permeability studies for melatonin and Flu-Na were conducted using nasal mucosa tissues obtained from the same pig (n = 3) and from three different pigs, respectively.

Tissues obtained from the same pig were used to evaluate the effects of different IPA concentrations and the DPBS control within a single experimental set. To ensure reproducibility, the entire experiment was independently repeated using tissues from three different pigs.

For each experimental set, three pieces of nasal mucosa tissue from the same pig were submerged in 3 mL of IPA at concentrations of 10%, 50%, or 100% for 30 min to induce graded levels of epithelial damage. A parallel control tissue sample from the same pig was submerged in 3 mL of DPBS under identical conditions. Following exposure, all tissues were thoroughly rinsed with DPBS to remove residual solvent and were immediately mounted in Franz diffusion cells for permeability testing.

### 3.3. Permeation Study Using Franz Diffusion Cells

Nasal mucosal permeability was evaluated using a Phoenix™ DB-6 Franz diffusion system (Teledyne Hanson Research, Los Angeles, CA, USA), equipped with a diffusion area of 1.0 cm^2^ and a receptor volume of 10 mL. The nasal mucosa was mounted between the donor and receptor chambers, with the epithelial side facing the donor compartment. DPBS was used as the receptor medium and maintained at 37 ± 0.5 °C under continuous stirring at 600 rpm. At predetermined time points (0.5, 1, 2, 3, 4, 5, 6, 7, and 8 h), 0.5 mL of sample was withdrawn from the receptor compartment and immediately replaced with fresh DPBS to maintain sink conditions [26].

#### 3.3.1. Permeation Study of Melatonin

The melatonin loading solution was prepared by dissolving an appropriate amount of melatonin powder in 5 mL of DPBS, stirring for 15 min, and filtering through a 0.2 µm nylon membrane filter (Fisher Scientific, Waltham, MA, USA). The resulting concentrations were 1008 μg/mL for the same-pig group, and 990, 1020, and 1070 μg/mL for the different-pig groups, respectively.

At the start of the experiment, 0.5 mL of melatonin loading solution was added to the donor compartment. Receptor samples collected at each time point were mixed with an equal volume of acetonitrile, vortexed, and centrifuged (AccuSpin Micro17, Fisher Scientific, Waltham, MA, USA) at 10,000× *g* for 20 min. The resulting supernatants were collected for HPLC analysis.

#### 3.3.2. Permeation Study of Flu-Na

The Flu-Na loading solution was prepared at a fixed concentration of 1 mg/mL by dissolving 25 mg of Flu-Na in 25 mL of DPBS using volumetric flasks. At the start of the experiment, 0.5 mL of Flu-Na loading solution was added to the donor compartment. Receptor samples collected at each time point were centrifuged (AccuSpin Micro17, Fisher Scientific, Waltham, MA, USA) directly at 10,000× *g* for 20 min. The supernatants were collected for microplate reader (SpectraMax iD5, Molecular Devices, San Jose, CA, USA) analysis.

### 3.4. Analytical Methods

Melatonin concentrations in receptor samples were quantified using a high-performance liquid chromatography system (Shimadzu Corporation, Kyoto, Japan). The instrumentation and chromatographic conditions were consistent with those reported in a previous study [52]. The method exhibited a linear response over the range of 0.79 to 50 µg/mL (r^2^ > 0.999), with a limit of detection of 0.081 µg/mL and a limit of quantification of 0.40 µg/mL. Data acquisition and analysis were performed using EZStart 7.2 software (Shimadzu Corporation, Kyoto, Japan).

Flu-Na concentrations were determined using a microplate reader (SpectraMax iD5, Molecular Devices, San Jose, CA, USA). A 100 μL aliquot of each supernatant was transferred into a well of a 96-well plate, and absorbance was measured at 492 nm. Quantification was based on a standard calibration curve prepared from serial dilutions of a 1 mg/mL Flu-Na stock solution, covering a range linearity of 0.4 to 25 µg/mL (r^2^ > 0.999). All measurements were performed in quadruplicate, with DPBS used as the blank.

### 3.5. Calculation and Statistical Analysis

The permeation curve is reported as percentage of drug permeated and was computationally corrected for the withdrawn volume using an Excel (Microsoft, Redmond, WA, USA) add-in program DDSolver 1.0 [53]. Papp were calculated as described in previous work [26].

To evaluate the minimum mean difference required to achieve statistical significance (two-tailed, *p* < 0.05) between two groups in an independent two-sample *t*-test, the following equation was used:

(1) $$Relative\;Minimum\;Detectable\;Difference\left(\%\right)=t_{\alpha /2,2n-2}\times \sqrt{2}\times \frac{CV}{\sqrt{n}}\times 100$$

where $t_{\alpha /2,2n-2}$ is the critical value of the *t*-distribution at a two-tailed significance level of 0.05 with 2n−2 degrees of freedom, CV represents the coefficient of variation, and n denotes the sample size of each group. This calculation assumes equal sample sizes and homogeneous variability between the two groups.

### 3.6. Numerical Simulation of Permeation Experiments

Numerical simulations were performed using COMSOL Multiphysics^®^ version 6.2 (COMSOL Inc., Burlington, MA, USA), following the methodology established in our previous study [26]. The simulations were based on Fick’s law of diffusion, describing solute concentration changes over time and space within a defined three-dimensional geometry. The “Transport of Diluted Species” physics interface was employed to simulate drug diffusion dynamics, while adsorption effects were incorporated to account for drug retention within the nasal mucosa.

Variations in mucosal thickness among samples can significantly influence calculated permeability values and obscure true differences between test compounds. To address this, the simulation model was first fitted to experimentally obtained permeation profiles to extract key transport parameters. These parameters were subsequently used to normalize each permeation curve to a standardized mucosal thickness of 0.80 mm. Thickness correction minimized the impact of variability and enabled more accurate and consistent comparisons across experimental conditions.

A detailed description of the modeling framework, boundary conditions, and underlying mathematical assumptions has been provided in our previous publication [26].

### 3.7. Measurement of Flu-Na UV Spectra and Log D at Different pH Values

#### 3.7.1. Flu-Na UV Spectra

A Flu-Na solution (20 µg/mL) was prepared in DPBS and adjusted to pH values ranging from 6.0 to 10.0 using sodium hydroxide or hydrochloric acid solutions. For each pH condition, the absorbance spectrum was recorded over the wavelength range of 300–600 nm using a microplate reader (SpectraMax iD5, Molecular Devices, San Jose, CA, USA).

#### 3.7.2. Flu-Na Log D

Equal volumes of *n*-octanol and DPBS were mixed and shaken at room temperature for 24 h using an incubator shaker (Lab-Line Instruments, Inc., Melrose Park, IL, USA) to achieve mutual saturation. The pre-saturated *n*-octanol and DPBS phases were then carefully transferred into separate centrifuge tubes and centrifuged (Centrifuge 5430 R, Eppendorf AG, Hamburg, Germany) at 5000× *g* for 20 min to ensure complete phase separation.

Flu-Na solutions were prepared at a final concentration of 1 mg/mL by dissolving Flu-Na in pre-saturated DPBS, followed by pH adjustment to approximately 6, 7, 8, 9, and 10 using sodium hydroxide or hydrochloric acid solutions. Flu-Na solutions at different pH values were prepared by dissolving Flu-Na in pre-saturated DPBS to a final concentration of 1 mg/mL, followed by pH adjustment to approximately 6, 7, 8, 9, or 10 using sodium hydroxide or hydrochloric acid solutions. For each pH condition, 2 mL of pH-adjusted Flu-Na solution was mixed with 10 mL of pre-saturated *n*-octanol in a glass vial and shaken at room temperature for 30 min to reach partitioning equilibrium. After shaking, the upper (*n*-octanol) and lower (DPBS aqueous) phases were separated by pipetting and then centrifuged again (AccuSpin Micro17, Fisher Scientific, Waltham, MA, USA) at 5000× *g* for 20 min to ensure clear phase separation.

The concentrations of Flu-Na in both phases were measured at 492 nm using the microplate reader (SpectraMax iD5, Molecular Devices, San Jose, CA, USA). Calibration curve standards were prepared using DPBS adjusted to the corresponding pH values to ensure accurate quantification under each condition. The log D was calculated as the base-10 logarithm of the concentration ratio between the *n*-octanol phase and the aqueous phase.

## 4. Conclusions

This study underscores the critical importance of addressing mucosal thickness variability in ex vivo nasal permeability studies. By integrating numerical simulation for post hoc thickness normalization, we achieved a substantial reduction in experimental variability, particularly for melatonin, thereby enhancing both data reliability and statistical power. These findings validate mucosal thickness as a significant confounding factor and support the implementation of computational correction methods to improve reproducibility across both intra- and inter-individual samples.

In contrast, Flu-Na, a widely used paracellular integrity marker, exhibited inconsistent permeability behavior that did not reliably correlate with mucosal thickness or chemically induced epithelial damage. The paradoxical decrease in Flu-Na permeability following mild IPA exposure suggests that chemical insult may induce non-linear or obstructive changes in the paracellular pathway, limiting its utility as a standalone marker for assessing mucosal barrier integrity under physiologically complex conditions.

Altogether, these results advocate for a paradigm shift in ex vivo nasal permeability assay design—one that incorporates structural correction through thickness normalization and emphasizes the context-specific selection of integrity markers. Adoption of these refinements is expected to improve experimental fidelity, optimize tissue utilization, reduce animal use, and enhance the translational relevance of ex vivo permeability models in nasal drug development.

## Figures and Tables

**Table 1:** Estimated minimum detectable differences according to CV and sample size.

Sample Size (n)	CV (%)	Relative Minimum Detectable Difference (%)
3	10	22.67
3	20	45.33
3	30	68.00
4	10	19.63
4	20	39.26
4	30	58.89
5	10	17.56
5	20	35.11
5	30	52.67

## Data Availability

Data is contained within the article and supplementary material.

## Supplementary Materials

The following supporting information can be downloaded at: https://www.mdpi.com/article/10.3390/ph18060889/s1, Table S1: Parameters estimation results from simulation.

## References

[1] Kola I., Landis J. (2004). Can the pharmaceutical industry reduce attrition rates?. Nat. Rev. Drug Discov..

[2] Sun D., Gao W., Hu H., Zhou S. (2022). Why 90% of clinical drug development fails and how to improve it?. Acta Pharm. Sin. B.

[3] Kirby-Smith C., Steenekamp J., Steyn D., Haasbroek-Pheiffer A., Hamman H., Hamman J. (2023). Intranasal Insulin Delivery: Microparticle Formulations Consisting of *Aloe vera* Polysaccharides for Advanced Delivery across Excised Olfactory and Respiratory Nasal Epithelial Tissues. Appl. Sci..

[4] Liu Y., Johnson M.R., Matida E.A., Kherani S., Marsan J. (2009). Creation of a standardized geometry of the human nasal cavity. J Appl. Physiol..

[5] Gizurarson S. (2012). Anatomical and histological factors affecting intranasal drug and vaccine delivery. Curr. Drug Deliv..

[6] Helander H.F., Fändriks L. (2014). Surface area of the digestive tract–revisited. Scand. J. Gastroenterol..

[7] DeSesso J.M., Jacobson C.F. (2001). Anatomical and physiological parameters affecting gastrointestinal absorption in humans and rats. Food Chem. Toxicol..

[8] Dahl R., Mygind N. (1998). Anatomy, physiology and function of the nasal cavities in health and disease. Adv. Drug Deliv. Rev..

[9] Merkus F.W., Verhoef J.C., Schipper N.G., Marttin E. (1998). Nasal mucociliary clearance as a factor in nasal drug delivery. Adv. Drug Deliv. Rev..

[10] Gizurarson S. (2015). The effect of cilia and the mucociliary clearance on successful drug delivery. Biol. Pharm. Bull..

[11] Haasbroek-Pheiffer A., Van Niekerk S., Van der Kooy F., Cloete T., Steenekamp J., Hamman J. (2023). In vitro and ex vivo experimental models for evaluation of intranasal systemic drug delivery as well as direct nose-to-brain drug delivery. Biopharm. Drug Dispos..

[12] Steyn J.D., Haasbroek-Pheiffer A., Pheiffer W., Weyers M., van Niekerk S.E., Hamman J.H., van Staden D. (2025). Evaluation of Drug Permeation Enhancement by Using In Vitro and Ex Vivo Models. Pharmaceuticals.

[13] Loftsson T., Konradsdottir F., Masson M. (2006). Development and evaluation of an artificial membrane for determination of drug availability. Int. J. Pharm..

[14] Henriques P., Bicker J., Silva S., Doktorovova S., Fortuna A. (2023). Nasal-PAMPA: A novel non-cell-based high throughput screening assay for prediction of nasal drug permeability. Int. J. Pharm..

[15] Radan M., Djikic T., Obradovic D., Nikolic K. (2022). Application of in vitro PAMPA technique and in silico computational methods for blood-brain barrier permeability prediction of novel CNS drug candidates. Eur. J. Pharm. Sci..

[16] Soriano-Meseguer S., Fuguet E., Port A., Rosés M. (2023). Suitability of skin-PAMPA and chromatographic systems to emulate skin permeation. Influence of pH on skin-PAMPA permeability. Microchem. J..

[17] Dargo G., Vincze A., Muller J., Kiss H.J., Nagy Z.Z., Balogh G.T. (2019). Corneal-PAMPA: A novel, non-cell-based assay for prediction of corneal drug permeability. Eur. J. Pharm. Sci..

[18] Yamashita S., Tanaka Y., Endoh Y., Taki Y., Sakane T., Nadai T., Sezaki H. (1997). Analysis of drug permeation across Caco-2 monolayer: Implication for predicting in vivo drug absorption. Pharm. Res..

[19] Sibinovska N., Zakelj S., Trontelj J., Kristan K. (2022). Applicability of RPMI 2650 and Calu-3 Cell Models for Evaluation of Nasal Formulations. Pharmaceutics.

[20] Bai S., Yang T., Abbruscato T.J., Ahsan F. (2008). Evaluation of human nasal RPMI 2650 cells grown at an air-liquid interface as a model for nasal drug transport studies. J. Pharm. Sci..

[21] Gerber W., Svitina H., Steyn D., Peterson B., Kotze A., Weldon C., Hamman J.H. (2022). Comparison of RPMI 2650 cell layers and excised sheep nasal epithelial tissues in terms of nasal drug delivery and immunocytochemistry properties. J. Pharmacol. Toxicol Methods.

[22] Wadell C., Bjork E., Camber O. (2003). Permeability of porcine nasal mucosa correlated with human nasal absorption. Eur. J. Pharm. Sci..

[23] Bartos C., Szabo-Revesz P., Horvath T., Varga P., Ambrus R. (2021). Comparison of Modern In Vitro Permeability Methods with the Aim of Investigation Nasal Dosage Forms. Pharmaceutics.

[24] Song J., Xu Z., Xie L., Shen J. (2025). Recent Advances in Studying In Vitro Drug Permeation Across Mucosal Membranes. Pharmaceutics.

[25] Nicolazzo J.A., Reed B.L., Finnin B.C. (2003). The effect of various in vitro conditions on the permeability characteristics of the buccal mucosa. J. Pharm. Sci..

[26] Zhao S., Zhao Y., Zuo J., Le T.S., Kerdsiri J., Waranuch N., Davies N.M., Loebenberg R. (2025). Evaluation of Drug Permeability Across Ex Vivo Nasal Mucosa: A Simulation-Based Approach to Minimize Thickness-Related Variability. J. Drug Deliv. Sci. Technol..

[27] Mehrer H. (2007). Diffusion in Solids: Fundamentals, Methods, Materials, Diffusion-Controlled Processes.

[28] Kumar N.N., Gautam M., Lochhead J.J., Wolak D.J., Ithapu V., Singh V., Thorne R.G. (2016). Relative vascular permeability and vascularity across different regions of the rat nasal mucosa: Implications for nasal physiology and drug delivery. Sci. Rep..

[29] Arora P., Sharma S., Garg S. (2002). Permeability issues in nasal drug delivery. Drug Discov. Today.

[30] Wadell C., Bjork E., Camber O. (1999). Nasal drug delivery—Evaluation of an in vitro model using porcine nasal mucosa. Eur. J. Pharm. Sci..

[31] de Araujo J.S.M., Augusto G.G.X., Pestana A.M., Groppo F.C., Rodrigues F.S.M., Novaes P.D., Franz-Montan M. (2024). Impact of Storage on In Vitro Permeation and Mucoadhesion Setup Experiments Using Swine Nasal Mucosa. AAPS PharmSciTech.

[32] Osth K., Grasjo J., Bjork E. (2002). A new method for drug transport studies on pig nasal mucosa using a horizontal Ussing chamber. J. Pharm. Sci..

[33] Leichner C., Baus R.A., Jelkmann M., Plautz M., Barthelmes J., Dunnhaupt S., Bernkop-Schnurch A. (2019). In vitro evaluation of a self-emulsifying drug delivery system (SEDDS) for nasal administration of dimenhydrinate. Drug Deliv. Transl. Res..

[34] Haasbroek-Pheiffer A., Viljoen A., Steenekamp J., Chen W., Hamman J. (2024). An Ex vivo Investigation on Drug Permeability of Sheep Nasal Epithelial Tissue Membranes from the Respiratory and Olfactory Regions. Curr. Drug Deliv..

[35] SAFETY DATA SHEET. Sigma-Aldrich. https://www.sigmaaldrich.com/CA/en/sds/sial/f6377?srsltid=AfmBOop0KjftBilbRx2MaZpl4wFn4amm3MMvTLw6X1YHBh4HJZ80_He1.

[36] Sakai M., Imai T., Ohtake H., Azuma H., Otagiri M. (1997). Effects of absorption enhancers on the transport of model compounds in Caco-2 cell monolayers: Assessment by confocal laser scanning microscopy. J. Pharm. Sci..

[37] Oba Y., Poulson S.R. (2012). Octanol-water partition coefficients (*K_ow_*) vs. pH for fluorescent dye tracers (fluorescein, eosin Y), and implications for hydrologic tracer tests. Geochem. J..

[38] Hurni M.A., Noach A.B., Blom-Roosemalen M.C., de Boer A.G., Nagelkerke J.F., Breimer D.D. (1993). Permeability enhancement in Caco-2 cell monolayers by sodium salicylate and sodium taurodihydrofusidate: Assessment of effect-reversibility and imaging of transepithelial transport routes by confocal laser scanning microscopy. J. Pharmacol. Exp. Ther..

[39] Ahishali B., Kaya M. (2020). Evaluation of blood-brain barrier integrity using vascular permeability markers: Evans blue, sodium fluorescein, albumin-alexa fluor conjugates, and horseradish peroxidase. Permeability Barrier: Methods and Protocols.

[40] Szabo A., Vollmar B., Boros M., Menger M.D. (2008). In vivo fluorescence microscopic imaging for dynamic quantitative assessment of intestinal mucosa permeability in mice. J. Surg. Res..

[41] McNamara N.A., Fusaro R.E., Brand R.J., Polse K.A., Srinivas S.P. (1997). Measurement of corneal epithelial permeability to fluorescein. A repeatability study. Investig. Ophthalmol. Vis. Sci..

[42] Clausen A.E., Bernkop-Schnurch A. (2000). In vitro evaluation of the permeation-enhancing effect of thiolated polycarbophil. J. Pharm. Sci..

[43] Clausen A.E., Bernkop-Schnurch A. (2001). Thiolated carboxymethylcellulose: In vitro evaluation of its permeation enhancing effect on peptide drugs. Eur. J. Pharm. Biopharm..

[44] Zambito Y., Zaino C., Burchielli S., Carelli V., Serafini M.F., Di Colo G. (2007). Novel quaternary ammonium chitosan derivatives for the promotion of intraocular drug absorption. J. Drug Deliv. Sci. Technol..

[45] Zambito Y., Uccello-Barretta G., Zaino C., Balzano F., Di Colo G. (2006). Novel transmucosal absorption enhancers obtained by aminoalkylation of chitosan. Eur. J. Pharm. Sci..

[46] Salade L., Wauthoz N., Goole J., Amighi K. (2019). How to characterize a nasal product. The state of the art of in vitro and ex vivo specific methods. Int. J. Pharm..

[47] Anderson J.M., Van Itallie C.M. (2009). Physiology and function of the tight junction. Cold Spring Harb. Perspect. Biol..

[48] Shah B., Khunt D., Misra M., Padh H. (2016). Non-invasive intranasal delivery of quetiapine fumarate loaded microemulsion for brain targeting: Formulation, physicochemical and pharmacokinetic consideration. Eur. J. Pharm. Sci..

[49] Patel R.B., Patel M.R., Bhatt K.K., Patel B.G. (2013). Formulation consideration and characterization of microemulsion drug delivery system for transnasal administration of carbamazepine. Bull. Fac. Pharm. Cairo Univ..

[50] Ohashi Y., Nakai Y., Koshimo H., Esaki Y., Ikeoka H., Horiguchi S., Teramoto K., Nakaseko H. (1988). Toxicity of isopropyl alcohol exposure on the nasal mucociliary system in the guinea pig. Environ. Res..

[51] Haasbroek-Pheiffer A., Viljoen A., Steenekamp J., Chen W., Hamman J. (2023). Permeation of Phytochemicals of Selected Psychoactive Medicinal Plants across Excised Sheep Respiratory and Olfactory Epithelial Tissues. Pharmaceutics.

[52] Zhao S., Le T.S., Davies N.M., Löbenberg R. (2025). Development of a Physiologically Relevant Simulated Nasal Fluid for In Vitro Dissolution Studies. Dissolution Technol..

[53] Zhang Y., Huo M., Zhou J., Zou A., Li W., Yao C., Xie S. (2010). DDSolver: An add-in program for modeling and comparison of drug dissolution profiles. AAPS J..

