# Supplementary material for: Improving Ex Vivo Nasal Mucosa Experimental Design for Drug Permeability Assessments: Correcting Mucosal Thickness Interference and Reevaluating Fluorescein Sodium as an Integrity Marker for Chemically Induced Mucosal Injury
Source: Pharmaceuticals (Basel). 2025 Jun 13;18(6):889. doi: 10.3390/ph18060889 (PMC12195738; doi:10.3390/ph18060889)
Supplement: Supplementary file 1 [file pharmaceuticals-18-00889-s001.zip › pharmaceuticals-3655035-supplementary.pdf]

Table S1. Parameters Estimation Results from Simulation.

		D_fit ($\times 10^{-10}$ m²/s)	K_fit ($\times 10^{-2}$)	Ep_fit	Objective
**Melatonin-Same Pig**	Melatonin T1_0.47 mm	1.18	1.00	1.08	0.1312
	Melatonin T2_0.72 mm	1.02	0.98	1.04	0.0491
	Melatonin T3_0.81 mm	1.18	1.00	1.00	0.1096
**Melatonin-Different Pigs**	Melatonin T1_0.46 mm	1.17	1.00	1.00	0.2346
	Melatonin T2_0.55 mm	1.28	1.01	1.02	0.1312
	Melatonin T3_0.82 mm	1.20	0.99	1.06	0.0371
**Flu Na-Same Pig**	Flu Na T1_0.75 mm	0.82	0.31	0.70	0.0061
	Flu Na T2_0.81 mm	0.83	0.28	0.74	0.0120
	Flu Na T3_1.05 mm	0.80	0.28	0.71	0.0061
**Flu Na-Different Pigs**	Flu Na T1_0.75 mm	1.23	0.28	0.76	0.0272
	Flu Na T2_0.83 mm	0.83	0.29	0.74	0.0195
	Flu Na T3_0.99 mm	1.85	0.33	0.61	0.0098
